# The Italian Association for Radiotherapy and Clinical Oncology (AIRO) position statements for postoperative breast cancer radiation therapy volume, dose, and fractionation

**DOI:** 10.1007/s11547-022-01563-9

**Published:** 2022-10-06

**Authors:** Icro Meattini, Isabella Palumbo, Carlotta Becherini, Simona Borghesi, Francesca Cucciarelli, Samantha Dicuonzo, Alba Fiorentino, Ruggero Spoto, Philip Poortmans, Cynthia Aristei, Lorenzo Livi

**Affiliations:** 1 Department of Experimental and Clinical Biomedical Sciences “M. Serio”, University of Florence, Viale Morgagni 50, 50134 Florence, Italy; 2 Radiation Oncology Unit, Oncology Department, Azienda Ospedaliero Universitaria Careggi, Viale Morgagni 50, 50134 Florence, Italy; 3 Radiation Oncology Section, University of Perugia and Perugia General Hospital, Perugia, Italy; 4 Radiation Oncology Unit of Arezzo–Valdarno, Azienda USL Toscana Sud Est, Arezzo, Italy; 5 Radiotherapy Unit, Azienda Ospedaliero Universitaria Ospedali Riuniti, Ancona, Italy; 6 Division of Radiation Oncology, IEO, European Institute of Oncology, IRCSS, Milan, Italy; 7 Radiation Oncology Department, General Regional Hospital F.Miulli, Acquaviva delle Fonti, Bari, Italy; 8 Department of Radiotherapy, Humanitas Clinical and Research Center, IRCSS, Rozzano, Milan, Italy; 9 Department of Radiation Oncology, Iridium Netwerk, Antwerp, Belgium; 10 Faculty of Medicine and Health Sciences, University of Antwerp, Antwerp, Belgium

**Keywords:** Breast cancer, Radiotherapy, Hypofractionation, Guidelines, Ultra-hypofractionation, Partial breast irradiation

## Abstract

Recent advances in non-metastatic breast cancer radiation therapy significantly reshaped our views on modern dose and fractionation schedules. Especially the advent of hypofractionation and partial breast irradiation defined a new concept of treatment optimization, that should strongly include both patient and tumour characteristics in the physician’s decision-making process. Unfortunately, hypofractionation for breast cancer radiation therapy needed long time to enter the routine practice during the last decades despite the level-1 evidence published over time. Hereby we present the Italian Association for Radiotherapy and Clinical Oncology (AIRO) Breast Cancer Group position statements for postoperative breast cancer radiation therapy volume, dose, and fractionation to harmonically boost routine clinical practice implementation following evidence-based data.

## Introduction

Recent advances in non-metastatic breast cancer radiation therapy significantly reshaped our views on modern dose and fractionations schedules. Especially the advent of hypofractionation and partial breast irradiation defined a new concept of treatment optimization, that should strongly consider both patient and tumour features in the decision-making process. In this framework, the European Society for Radiotherapy and Oncology Advisory Committee in Radiation Oncology Practice (ESTRO-ACROP) consensus recommendations on patient selection and dose and fractionation for external-beam radiotherapy in early breast cancer have been recently released [1].

To facilitate and enhance the breast oncologist’s community harmony, the Italian Association for Radiotherapy and Clinical Oncology (AIRO) Breast Cancer Group felt the need for a prompt reaction to integrate this level-1 evidence in the routine clinical practice. Hereby we present the position statements for postoperative breast cancer radiation therapy volume, dose, and fractionation.

## Position statements

- *Hypofractionation is considered standard of care for all indication of external-beam postoperative breast cancer radiation therapy, regardless of the number and size of target volumes and breast reconstruction*. Hypofractionation is standard of care both for invasive and ductal carcinoma in situ of the breast. There is no reason to prescribe irradiation schedules using more than 15–16 fractions [1–6].
- *50 Gy in 25 fractions is no longer considered being standard of care*. It should be restricted to highly selected cases, such as concomitant chemoradiation and hyperthermia to enhance the radio-sensitisation effects of the combined systemic or local agents [3, 4].
- *5-fraction whole breast and/or chest wall irradiation without reconstruction (26 Gy in 5 fractions) is considered standard of care*. This schedule it is not to be considered experimental and should be considered the preferred option especially (but not exclusively) in patients fulfilling the inclusion criteria of the FAST-Forward trial [1, 7–9].
- *Moderate hypofractionation should be offered for regional nodal irradiation* [1, 3, 4, 6]. Postmastectomy hypofractionated radiation therapy is non-inferior to and had similar toxicities to conventional fractionated radiation therapy in patients with high-risk breast cancer [6].
- *Partial breast irradiation is standard of care in selected patients affected by early breast cancer*. Especially (but not exclusively) in case of suitable features presence accordingly to the ESTRO-ACROP 2022 Consensus statements, partial breast irradiation should be preferred over whole breast irradiation [1, 10–18].

## Discussion

The AIRO felt the strong needs for sake of clarity to endorse the recently published Consensus statements released in 2022 by the ESTRO-ACROP initiative [1]. Hypofractionation for breast cancer radiation therapy needed too much time to enter the routine clinical practice during the last decades despite the level-1 evidence published over time [1–6].

Hypofractionation for breast cancer radiation therapy passed through a long-lasting debate about its safety and efficacy, although there are no economic [19], radiobiologic [20], nor clinic reasons [2, 5, 21] to justify these uncertainties. And this fact was probably caused by several heterogeneous factors, such as shortage of experience in hypofractionation, minimal resources for quality assurance in radiotherapy, inadequate support to change, and reimbursement policies [1]. However, we strongly believe that one of the crucial boosts to harmonically implement evidence-based data in the routine practice of a Country is represented by a clear, transparent, and strong position statement released by the national Society of the leading discipline.

Endorsing the European ESTRO-ACROP initiative [1], in line with the UK Breast Radiotherapy Consensus Working Group [9], we would like to enhance and reinforce the evidence supporting hypofractionation for all the indications of external-beam postoperative radiation therapy for non-metastatic breast cancer, including whole and partial breast, chest wall with or without reconstruction, and regional nodal irradiation. If moderate hypofractionation (40–42.5 Gy in 15–16 fractions) represents the standard of care for all the above-mentioned indications, ultra-hypofractionation (26 Gy in 5 fractions) should be considered standard of care for whole breast irradiation and chest wall irradiation without reconstruction. Conversely, further data are awaited to confirm the recommendation in favour of ultra-hypofractionation concerning chest wall with reconstruction and regional nodal irradiation (Table 1).

**Table 1:** Volume, dose, fractionation AIRO breast cancer group recommendations

	50 Gy in 25 fractions	40–42.5 Gy in 15–16 fractions	26 Gy in 5 fractions
Whole breast irradiation	Not recommended	Recommended°	Recommended°
Partial breast irradiation	Not recommended	Recommended°	Recommended°*
Chest wall irradiation without reconstruction	Not recommended ^^^	Recommended°	Recommended
Chest wall irradiation with reconstruction	Not recommended ^^^	Recommended°	Not recommended
Regional nodal irradiation	Not recommended ^^^	Recommended°	Not recommended

^^^ Except for highly selected cases, such as concomitant chemoradiation and hyperthermia to enhance the radio-sensitisation effects of the combined systemic or local agents

° Gold standard schedule

^*^ Gold standard for partial breast irradiation (26-30 Gy in 5, once-daily, consecutive fractions)

External-beam partial breast irradiation should be preferred over whole breast irradiation in case of clearly identified suitable features (Table 2) [1]. In these selected patients, partial breast irradiation using once-daily, consecutive fractions, schedule (40 Gy in 15 fractions or 26-30 Gy in 5 fractions) warrants equivalent disease control and a favourable safety toxicity profile [11, 14–17].

**Table 2:** Partial breast irradiation suitable patient selection criteria

Factor	Selection criteria
Patient-related	Age 50 years or more
Tumour-related	Luminal-like subtypes small tumour (≤ 3 cm)
	Clear surgical margins (> 2 mm)
	Node negative (including isolated tumour cells)
	Absence of lymph vascular space invasion
	Non-lobular invasive carcinoma
	Tumour grade 1–2
	Low-to-intermediate grade DCIS, sized ≤ 2.5 cm, clear surgical margins (≥ 3 mm)
	Unicentric or unifocal
Treatment-related	No use of primary systemic therapy and neoadjuvant chemotherapy

*DCIS* ductal carcinoma in situ

Fast implementation of short course radiation therapy schedules will warrant equity of access for all our patients. At the same time, benefits and risks, including uncertainties, of all available cancer treatments should be always discussed and shared with our patients, warranting an adequate counselling on the best evidence-based radiation therapy.
